# Peritumoral Brain Zone in Astrocytoma: Morphology, Molecular Aspects, and Clinical Manifestations (Review)

**DOI:** 10.17691/stm2024.16.2.08

**Published:** 2024-04-27

**Authors:** A.S. Grishin, K.A. Achkasova, L.S. Kukhnina, V.A. Sharova, M.V. Ostapyuk, K.S. Yashin

**Affiliations:** Pathologist, Pathological Anatomy Unit, University Clinic; Privolzhsky Research Medical University, 10/1 Minin and Pozharsky Square, Nizhny Novgorod, 603005, Russia; Assistant, Department of Pathological Anatomy; Privolzhsky Research Medical University, 10/1 Minin and Pozharsky Square, Nizhny Novgorod, 603005, Russia; Junior Researcher, Laboratory of Optical Coherence Tomography, Institute of Experimental Oncology and Biomedical Technologies; Privolzhsky Research Medical University, 10/1 Minin and Pozharsky Square, Nizhny Novgorod, 603005, Russia; Student; Privolzhsky Research Medical University, 10/1 Minin and Pozharsky Square, Nizhny Novgorod, 603005, Russia; Student; Privolzhsky Research Medical University, 10/1 Minin and Pozharsky Square, Nizhny Novgorod, 603005, Russia; Neurosurgeon, Neurosurgery Unit, University Clinic; Privolzhsky Research Medical University, 10/1 Minin and Pozharsky Square, Nizhny Novgorod, 603005, Russia; Assistant, M.V. Kolokoltsev Department of Traumatology, Orthopedics, and Neurosurgery; Privolzhsky Research Medical University, 10/1 Minin and Pozharsky Square, Nizhny Novgorod, 603005, Russia; MD, PhD, Neurosurgeon, Neurosurgery Unit, University Clinic; Privolzhsky Research Medical University, 10/1 Minin and Pozharsky Square, Nizhny Novgorod, 603005, Russia; Assistant, M.V. Kolokoltsev Department of Traumatology, Orthopedics, and Neurosurgery; Privolzhsky Research Medical University, 10/1 Minin and Pozharsky Square, Nizhny Novgorod, 603005, Russia; Oncologist, Outpatient Department; Nizhny Novgorod Regional Oncologic Dispensary, 11/1 Delovaya St., Nizhny Novgorod, 603163, Russia

**Keywords:** glioma, peritumoral brain zone, low-grade astrocytomas, glioblastoma, epilepsy

## Abstract

A peritumoral brain zone is an area between a tumor and nontumorous brain tissue with tumor cell infiltration. The identification of this area is sufficiently difficult due to the lack of clear morphological or some other criteria. Besides, its dimensions may vary considerably. In the present review, we have analyzed the available data on the morphological structure and metabolism of peritumoral zone in astrocytomes, and considered the main molecular and genetic aspects and clinical manifestations.

Exploration of the peritumoral zone is of great importance for determining the extent of resection to prevent recurrence and to reveal the causes and mechanisms of continued tumor growth.

## Introduction

Gliomas (astrocytomas) represent a group of various neoplasms, which develop from glial cells of the central nervous system (CNS) [1, 2], and is the most prevalent form among malignant tumors of the CNS. Malignant high-grade gliomas occur most commonly: grade III and IV account for 85% of cases. Five-year survival rates vary depending on the malignancy grade and are 82% for grade I, 54% for grade II, 22% for grade III, and 3% for grade IV [3, 4]. The average survival rate in glioblastoma is about 14.5 months [5, 6].

Treatment of patients with astrocytomas is combined and includes radical save resection, radiation and drug therapy depending on the tumor molecular profile. Even if radical save resection is performed and all standard protocols are fulfilled, its growth is usually resumed, with the continued growth occurring directly in the area of the primary tumor in 90% of cases [7]. It is for this reason that exploration of peritumoral area is of great importance for identifying the causes and mechanisms of continued tumor growth and determining optimal extent of resection to prevent recurrence and increase patient survival rate [8–10].

The literature search was carried out using PubMed/ MEDLINE, eLIBRARY.RU, Google Scholar systems and the following key words and their combinations: peritumoral brain zone, peritumoral brain area, astrocytoma microenvironment, peritumoral area in astrocytomas.

## Defining glioma peritumoral area

The peritumoral zone is considered to occupy an area between a tumor and normal tissue with tumor cell infiltration [11, 12]. However, there is no agreement regarding exact criteria to define peritumoral zone and its borders [13]. As compared to a healthy brain tissue, this area is characterized by a set of molecular, biochemical, radiological, and cellular specific features. Tumor cells in the peritumoral zone possess high invasive capacity in contrast to the cells of the tumor core [7, 14] and play a key role in tumor recurrence [15].

Identification of peritumoral area by neuroimaging is difficult. Presently, this zone is described as hypointensive on contrast-enhanced T1-WI and hyperintensive on T2-WI. If defined in this way, its dimensions on the standard MRI sequences may be large enough. This has been confirmed by some investigations. Thus, Glas et al. [16] have reported about the presence of tumor cells at a distance of several centimeters from the tumor. Aubry et al. [17] and Csutak et al. [18] have established that the area with characteristic molecular, radiological, and cellular features may extend over 1 cm from the tumor margin. Since it was not possible to delineate clearly the changed and healthy brain tissue, the term “edema– infiltration area” [19, 20] has been proposed.

## Morphological aspects of glial peritumoral zone

**Figure 1.**
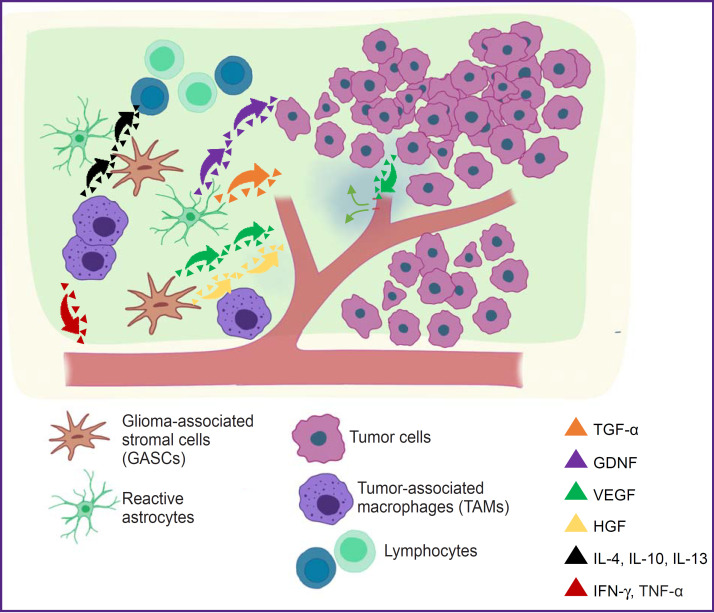
Morphological aspects of the peritumoral glioma zone The peritumoral area is presented by a wide diversity of cells: reactive astrocytes, which promote indirect glioma spreading by BDNF, TGF-α, and GDNF synthesis; tumor-associated macrophages, which, depending on the subtype, are involved in the suppression of the tumor growth releasing IFN-γ, TNF-α cytokines and provide tumor invasion releasing IL-4, IL-10, IL-13; glioma-associated stromal cells, which promote tumor invasion by VEGF synthesis and especially HGF; lymphocytes

A higher cell density is usually observed in the peritumoral zone than in the unchanged brain tissue. The MRI-derived cellularity index has shown a negative gradient in the form of the decreased number of cells from the tumor center to the peritumoral area [21]. The cell composition in the peritumoral zone is highly diverse and may vary quantitatively from case to case but at the same time it has a substantial similarity to both the tumor and the brain tissue [22–26] (Figure 1). A significant part of the peritumoral zone is presented by astrocytic and (to the less extent) oligodendroglial components. The majority of these cells show reactive properties typical for gliosis. Reactive astrocytes are highly proliferative, they are metabolically active and more intensively interact with their microenvironment. These astrocytes are indirectly involved in glioma spreading, including a metabolic way, by synthetizing multiple cytokines: brain-derived neurotrophic factor (BDNF), transforming growth factor α (TGF-α), sphingosine-1-phosphate, glial cell line-derived neurotrophic factor (GDNF) [7, 27–29].

The vascular part is presented by endothelium including stem endothelial cells and pericytes. The microvascular component of the peritumoral zone usually proliferates more actively, the vascular density is higher as compared to the unchanged tissue but it does not have deformations typical of tumoral vessels in the form of chaotic, anastomosing, incompetent vascular channels with formation of chains or glomeruloid structures [11]. In hese conditions, a greater permeability of the vascular wall and blood–brain barrier impairment are noted, which together with the increased spreading capacity of the tumor lead to the development of peritumoral edema [18]. High permeability pathogenesis is associated with the disruption of the tight junction integrity of endothelial cells in the microvessel walls due to protein phosphorylation under the influence of the vascular endothelial growth factor (VEGF) secreted by tumor cells. This mechanism is typical for all gliomas [22]. The immune component bears mainly reactive character, and is presented by macrophages of the resident microglia as well as multiple low-specificity cells of myeloid origin such as neutrophils, histiocytes, plasmocytes, eosinophils, lymphocytes. The cells of macrophagial nature are, as a rule, more active and possess increased phagocytosis, while their cytockeleton is structurally changed. Tumor-associated macrophages (TAMs) also play an important role in maintaining the tumor growth, have a prognostic value, and are considered as a target for adjunctive methods of glioma therapy [30–32]. TAMs are usually divided into polarized macrophages M1 and M2 activated by interferon gamma (IFN-γ) and IL-4, respectively. Macrophages M1 are capable of releasing proinflammatory cytokines (IFN-γ), presenting antigens to immune cells, and phagocytizing tumor cells. Meanwhile, macrophages M2 possess immunosuppressive action and provide tumor progression and invasion releasing anti-inflammatory cytokines (IL-4, IL-13). An increased number of regulatory T-cells is noted, especially at the expense of CD8+ population, which is much less presented in the tumor tissue in contrast to CD4+ cells, with which the parity is observed [26].

Another key feature of the peritumoral zone is the presence of glioblastoma-associated stromal cells (GASCs). These cells have the same characteristics as mesenchymal diploid stem cells without genomic changes typical of gliomas. GASC origin has not been fully established, however, functionally and phenotypically they resemble tumor-associated fibroblasts. GASCs are likely to actively interact with tumor cells being involved in the accelerated tumor spreading by releasing VEGF and especially hepatocyte growth factor (HGF). In this case, two microenvironments are distinguished in the peritumoral area: with and without glioma-associated stromal cells. Thus, the predominant variant of microenvironment after resection determines the probability and speed of the reccurrence [7, 23].

Frequently, the tumor cells are located directly in the peritumoral zone, they are more invasive and proliferatively active than the cells of the tumor core, have some phenotypic differences including the immunohistochemical profile [16, 33–35].

## Structural changes of the peritumoral white matter occurring in glial neoplasms

**Figure 2.**
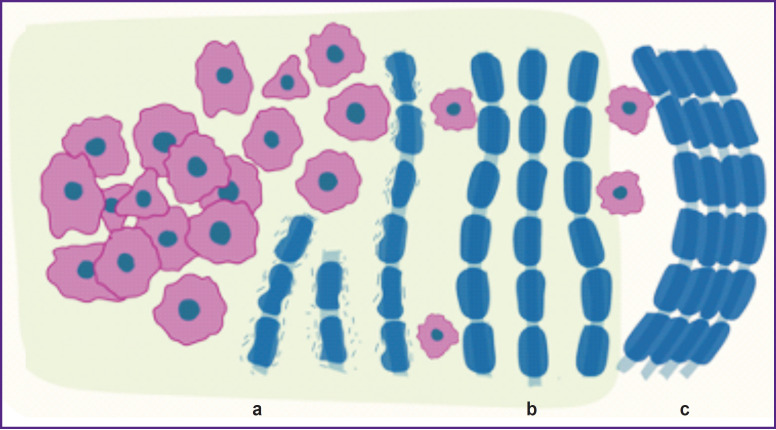
Structural changes of the white matter in brain tumors Partial demyelination of the nerve fibers (a) is present directly in the region of the tumor-infiltrated brain white matter. Fiber displacement may be observed as separation of the conductive pathways due to edema (b), and as a compaction (c) due to the direct growth of the tumor node

One of the defining characteristics of glial tumors is their fast invasive growth into the surrounding brain tissue through migration along the fibers of the white matter, growth near the neurons, and penetration along the blood vessels and meninges [36]. This feature differentiates glial tumors from metastatic intracranial neoplasms, which disseminate through hematogenic and lymphogenic ways only [37, 38], and also from benign tumors, which push aside the nerve tissue not invading it [39]. Glial tumors are known to develop often in the white matter and prefer metastasizing across the entire brain using myelinated fibers as a framework for cell migration. Besides, using this pathway, the tumor may disseminate to the contralateral hemisphere strictly along the fibers of the corpus collosum [40, 41]. Alterations in the white matter tissue occurring with the growing tumor may be classified as edema, infiltration, dislocation, and destruction of myelinic fibers [3, 42] (Figure 2). Demyelination of the nerve fibers should be separately highlighted.

Edema is of vasogenic origin and develops due to impairment of vascular wall integrity. The tumor cells, migrating along the vessels, push astrocyte processes away from the vascular wall thereby destroying perivascular basement membrane and breaking blood–brain barrier, which results in the increased permeability of the vascular wall, penetration of blood plasma into the brain parenchyma, and edema formation [43]. Displacement of fibers may lead either to their compaction (Figure 2 (c)) or separation of the pathways (Figure 2 (b)) for two main reasons. In the first case, displacement occurs as a result of the direct growth of the tumor core. The low-grade glial cells are known to cause displacement of the brain parenchyma without severe damage to the fibers [44]. The second reason is the above mentioned growing edema of the brain tissue. A key factor playing the most important role in the infiltrative growth of glial tumors within the peritumoral zone is an extracellular matrix (ECM). ECM in the brain tissue differs from the ECM of the majority of organs in composition and makes up only about 20% of the brain tissue volume [45, 46]. Parenchymal ECM is formed from glycosaminoglycans, hyaluronic acid in particular, proteoglycans without collagen molecules and coupling proteins. Basement membranes, like in other organs, consist of collagen, glycoproteins, and adhesion molecules.

Hyaluronic acid attracts special attention as it performs a number of functions in normal and pathologically changes brain tissues. Thus, in normal conditions hyaluronic acid, being part of ECM, maintains tissue homeostasis, biomechanical integrity, as well as the tissue structure. In case of malignant tumor, the content of hyaluronic acid significantly increases causing enhanced proliferation, spreading, and invasion of the tumor cells [47, 48]. This compound promotes actively tumor growth owing to the opening of the spaces for tumor cell migration [49–51]. In order to penetrate these tiny spaces, the tumor cells remodel their cytoskeleton and volume, which is achieved by the joint action of ion channels and myosin protein [52, 53]. Additionally, in the process of tumor invasion, active changes occur in ECM under the action of various enzymes, which destroy some ECM components facilitating tumor cell migration. Among the diversity of enzymes involved in the ECM restructuring (proteases, glycosides, hyaluronidases), separate attention should be payed to metalloproteases, since they are directly associated with tumor invasion along myelinated fibers.

However, the white matter also contains the components possessing protective antitumor effect. The CNS myelin is known to contain protein inhibitors blocking regeneration of axons and spreading of astrocytes and fibroblasts. Besides, these compounds slow down tumor cell invasion in low-grade astrocytomas, whereas high-grade gliomas are capable of overcoming the inhibiting effects of myelin [54, 55]. The latter is achieved by secreting the metalloprotease enzymes by the tumor cells destroying protein inhibitors, which results in migration of the tumor cells along the myelinated fibers. A higher rate of nervous tissue infiltration in high-grade gliomas, in contrast to low-grade ones, is probably associated with this process as well as the resistance of the tumor cells to ionized radiation effect in the course of radiation therapy [56].

Structural changes in myelin fibers consist in primary damage to myelin sheath and neurons, and oligodendrocyte death. It has also been found that tumor stem cells are located mainly near those areas of the nerve fibers where myelin has disappeared. Wang et al. [57] believe that continuous transmission of nerve impulses along axons maintains the vital activity of tumor cells and stimulates their further migration.

Damage to neurons and glias may be due to mechanical compression by the tumor, impaired nerve cell nourishment, and also secretion of exotoxins causing neuronal death and interrupting nerve impulse conduction [58]. Neuron death involves subsequent destruction of nerve fibers. Impairment of glial cell functions is a consequence of their active involvement into the production of a large amount of cytokines, metabolites, and signaling molecules affected by tumor cells. As a result, glial cells appear incapable of maintaining brain homeostasis and providing adequate nutrition for neurons [59].

Thus, tumor cells in the peritumoral zone influence all components of the white matter, including myelin fibers, inducing the described-above structural changes in the tissue of the white matter.

## Metabolism

The peritumoral area is characterized by metabolic and biochemical changes (manifesting itself in inflammation), amino acid transport disorder, and neurotransmitter abnormalities [60–62]. As compared to the healthy brain tissue, malignant tumors demonstrate an increased demand for amino acids, which enhances the work of amino acid carriers and accelerates metabolism [63]. According to one of the studies [64], the decreased pH level caused by tumor hypoxia is observed in the peritumoral zone leading to structural alterations in glial cells and their reactive proliferation. Metabolic transformations are accompanied by the disorder of sodium, potassium, zinc, and copper ion exchange in the extracellular space, which enhances neuronal excitability [64]. Significant changes are noted in the regulation of glutathione and enzymes affecting antioxidant cell defense. The peritumoral area is characterized by a higher expression level of receptors, amino acids, activators of transcription factors, markers of proliferation and invasion in comparison with the tumor tissue [22].

Alterations in the lipid exchange are also observed in the peritumoral area. The activity of hormone-dependent isoenzymes stearoyl coenzyme A desaturase (SCD) and fatty acid desaturases (FADS1 and FADS2) is significantly higher in the peritumoral zone than in the tumor center designating a more active biosynthesis of monounsaturated fatty acids and polyunsaturated fatty acids [65].

To visualize metabolic changes in the peritumoral area, PET technique may be used since it makes it possible to detect increased levels of pH and lactate, reduction of blood flow relative to the tumor tissue [66, 67]. In some investigations [8, 68], proton magnetic resonance spectroscopy has found an essential difference (>1.31) between the ratio of Cho (choline) and NAA (N-acetyl aspartate) in the peritumoral and contralateral regions.

## Molecular genetic profile

Large genomic changes, which are usually similar to the central part of the tumor, are observed in the perifocal area. Thus, a gain of chromosome 7 with amplification of the epidermal growth factor receptor (EGFR), deletion of chromosome 10, PTEN abrogation, CDKN2A/2B loss on chromosome 9 are noted in the peritumoral zone of gliablastomas and some astrocytomas [69]. High expression of some insufficiently studied markers is also typical for this area: Neuromedin B, HIST2H2AA, transcriptional regulator ID3. Also noted are increased expression of genes encoding proteins important for cell mobility (alpha-dystrobrevin (DTNA), CD99, and VCAM-1) and increased activity of the markers responsible for immunosuppression (for example, KLRC1 receptor) [70].

Part of the molecular genetic changes has a more specific character in comparison with the tissue in the tumor center. For example, cells with high expression of heterodimer CD98 and light chain LAT1 are found in the peritumoral zone, which is typical for glial stem cells [71]. Besides, a high expression of genes associated with neurogenesis such as *MAL*, *MOG*, *MAG*, and *MOBP* is observed in the peritumolral zone, which is highly likely caused by activization of the primary neuroparenchyma [72].

The level of IL-8 expression together with the complement receptors CXCR1/CXCR2 is traced in the specific groups of CD4+ T-lymphocytes, which play a key role in immunosuppression of the peritumoral zone [73].

Yang et al. [74] report in their study about four specific biomarkers, *ERMN*, *MOBP*, *PLP1*, *OPALIN* genes, whose expression is associated with quickly progressing gliomas in elderly people.

Due to a marked effect of *VEGFA*, *VEGFC*, and *VEGFD* genes, complement receptors VEGFR1, VEGFR2, VEGFR3, and HIF-1α, an increased vascular density is noted both in the peritumoral zone and in the tumor itself. At the same time, a number of studies [14, 75] have established particularly high expression of *VEGF* exactly in the peritumoral zone.

Among the differentially expressed genes, *UBE2C*, *NUSAP1*, *PBK*, *IGFBP2*, *SERPINA3* may be referred to the most active genes, which are involved in the growth and proliferation of the cells in many malignant tumors [26].

A number of the key proteomic components seem to be also activated precisely in the peritumoral zone, for example, H3F3A [76]

It should be kept in mind that multiple microRNAs have a specific profile on the glioma periphery and are likely to play their role in the majority of aspects associated with tumor progression [77]. The *ERBB2* gene has been established to be associated with radiosensitivity and to facilitate the infiltrative glioma growth [42, 78]. The *SERPINA3* gene, whose expression increases in case of astrocytoma progression, also play an essential role in the processes of proliferation, invasion, and formation of radioresistance [79]. Genes whose expression in the tumor zone is always increased, for example, *TOPORS*, are also encountered [11, 23, 60, 65].

Increased invasion of the tumor cells into the normal tissue occurs under the action of TGF-α, sphingosine-1-phosphate, stromal cell-derived factor 1 (SDF1/CXCL12), and GDNF, which are encoded by the genes with increased expression and are in the peritumoral brain zone promoting tumor mass enlargement [7, 80–83].

In one of the last investigations [84], genome landscape of the gliablastoma center was compared with that in the peritumoral area. The results appeared controversial: the similarity of the genome was great for part of the patients, whereas for some tumors, an individual genome of the peritumoral area and tumor center was revealed.

## Clinical manifestations. Epilepsy

The peritumoral brain area often plays a leading role in formation of neurological deficit. Neuronal and glial functional impairment as well as formation of specific microenvironment in the peritumoral zone determine its high epileptogenic potential [85–88]. This has been confirmed by the results of electrographic examinations demonstrated seizure occurrence in the immediate vicinity from the tumor [67, 89].

The development of epileptic seizures caused by the increase of glutamate concentration has been shown on the glioma model in mice. The growth of glutamate concentration in the peritumoral zone is connected with the activity of the system x_c_^−^, cystine/glutamate transporter. This transporter imports cysteine for antioxidant glutation synthesis, and cysteine absorption in this case is connected with glutamate release [90]. While examining patients with tumor-associated epilepsy, it has been found that glutamate concentration and expression of the cystine/glutamate transporter system were increased in the tumor samples and peritumoral area [91].

Elevated excitability of neurons in the peritumoral area is also associated with the mechanism of GABA inhibition impairment. GABA is an inhibitory mediator in the CNS, but changes in glutamatergic signal transmission in the neurons of the peritumoral area turn GABA from inhibitory to exciting neuromediator [92, 93]. One of the mechanisms of suppressing inhibitory GABA function is linked to the alterations in chlorine ion concentration. NKCC1 and КCC2 are the main regulators of ion concentration: NKCC1 increases, while KCC2 reduces the level of chlorine ions. Expression of NKCC1 and KCC2 in the glioma peritumoral area is higher than in the normal brain tissue [94].

Depending on the tumor localization, the types of epileptic seizures also change. For example, tumors located in the posterior parts of the anterior, inferior, and medial frontal gyri cause partial seizures, tumors in the right temporal lobe increase the risk of the complex partial seizure, while damage to the left premotor area may result in generalized seizures [95].

Structural MRI can visualize the tumor mass and peritumoral edema, however, it is not able to identify functional and metabolic disturbances associated with epilepsy development [60].

## Conclusion

The morphological structure of the peritumoral zone differs significantly from the healthy brain and tumor tissue. This area is heterogenous in its cell composition, possesses unique biochemical and molecular genetic characteristics. Taking into account multiple unsuccessful attempts to affect tumor cells, researchers pay now much attention to the study of the peritumoral zone. An exact definition of this area is necessary especially from the biological point of view for establishing neuroimaging criteria to visualize the borders of the tumor together with the peritumoral area as a source of continued growth. Extended knowledge on neuroimaging characteristics of peritumoral zone will allow the specialists to assess the possibility of supramarginal astrocytoma resection with a wide coverage of the peritumoral zone. New ways of the local tumor growth control may be also proposed including the development of drugs for local and systemic action able to prevent malignant transformation of the peritumoral area into the actively growing tumor.
